# UAV rapidly-deployable stage sensor with electro-permanent magnet docking mechanism for flood monitoring in undersampled watersheds

**DOI:** 10.1016/j.ohx.2022.e00325

**Published:** 2022-06-10

**Authors:** Corinne Smith, Joud Satme, Jacob Martin, Austin R.J. Downey, Nikolaos Vitzilaios, Jasim Imran

**Affiliations:** a University of South Carolina, Department of Mechanical Engineering, Columbia, SC, United States; b University of South Carolina, Department of Physics and Astronomy, Columbia, SC, United States; c University of South Carolina, Department of Civil Engineering, Columbia, SC, United States

**Keywords:** stage, flood monitoring, rapidly-deployable, UAV, water height, ultrasonic sensor, UAV, Unmanned Aerial Vehicle, EPM, Electro-permanent magnet

## Abstract

The availability of historical flood data is vital in recognizing weather-related trends and outlining necessary precautions for at-risk communities. Flood frequency, magnitude, endurance, and volume are traditionally recorded using established streamgages; however, the material and installation costs allow only a few streamgages in a region, which yield a narrow data selection. In particular, stage, the vertical water height in a water body, is an important parameter in determining flood trends. This work investigates a low-cost, compact, rapidly-deployable alternative to traditional stage sensors that will allow for denser sampling within a watershed and a more detailed record of flood events. The package uses a HC-SR04 ultrasonic sensor to measure stage, onboard memory for recording flood events, and an electropermanet magnet (EPM) to enable Unmanned Aerial Vehicle (UAV) deployments. Optional modules for solar panels and wireless communication can also be added to extend package longevity or allow wireless control of the EPM. The stage sensor package was found to have a range of 0.02 to 4 m with a 6.9 mm accuracy and capable of a 6.4 day long deployment. With the total cost of production at 271.37 USD, it is a cheaper and more flexible alternative to traditional stage sensors that will enable dense sensor networks and rapid response to flooding events.

## Graphical abstract

**Figure:**
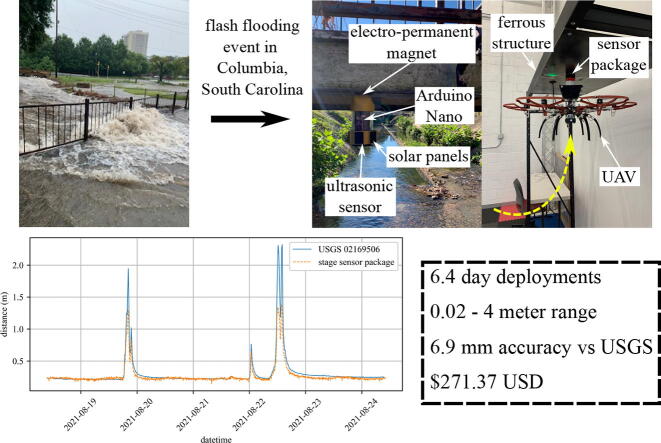

**Specifications table**

**Table:** 

Hardware name	Stage sensor package	
Subject area	Environmental, planetary and agricultural sciences	
Hardware type	Field measurements and sensors	
Closest commercial analog	USGS rapid deployment gage	
Open source license	CC-BY Attribution 4.0 International	
Cost of hardware	$271.37	
Source file repository	https://doi.org/10.17605/OSF.IO/A874U	

## 1 Hardware in Context

Flooding is on average the second deadliest weather-related hazard in the United States, causing 57 fatalities and inflicting over $1 billion in crop and property damage in 2020 alone [1]. Flash floods are characterized by intense, nucleated rainfall that drains into a common basin, usually too small to accommodate the volume of runoff generated by the localized storm. The swiftness and severity of the rainfall results in very sudden flooding, even if the rainfall is further upstream from the basin [2]. Built environments have become especially susceptible to flooding, as the increase in both rainfall events and impervious surfaces in heavily populated areas increase the occurrence of urban flooding [3]. For example, the Rocky Branch Watershed is an urban watershed located in Columbia, South Carolina, and is 97% developed with a high percentage of impervious surfaces [4].

Recent work at the regional level using a peaks-over-threshold approach to analyze flood magnitude and frequency trends has found that the magnitude of flood peaks has remained relatively consistent, however the frequency of historic flood events has increased significantly [5]. Frequent minor flooding, called nuisance flooding, has been found to have socioeconomic impacts on communities comparable to those incurred by extreme flooding, particularly for coastal communities [6]. On a larger scale, data from 375 streamgages across the contiguous United States was analyzed and revealed fragmented patterns of flood change that vary widely between geographical regions, with no real evident trends over large portions of the US [7]. The results of this study also conclude that even intraregional watersheds can exhibit high variability in trends concerning flood frequency, magnitude, duration, and volume. Due to this fragmentation, denser sampling within watersheds may provide a clearer picture of flood behavior on the regional scale. An extensive record of flood frequency and magnitude is crucial in planning for extreme disasters, assessing possible damages inflicted by nuisance flooding, and understanding flood trends within an area.

Stage is the vertical position of the water surface relative to an arbitrarily defined datum, and is a vital parameter in monitoring flood events. The United States Geological Survey (USGS) is a government agency that has established streamgages all throughout the US that collect data concerning weather events such as rainfall, water quality, and stage [8]. A USGS stage sensor can come in a variety of forms, including stilling wells, bubble gages, ultrasonic, and radar sensors. Stilling wells are typically 1.2 meters in diameter and operate by allowing stream water to enter through a pipe so that the water inside the well is at the same level as the water surface of the water body [9]. The water level inside the well is then measured using a float, pressure, ultrasonic, or optic sensor, and the data is logged electronically for a set time interval [10].

Bubble gages are a cheaper alternative to stilling wells [11]. These gages determine stage using pressure differences. A long tube is submerged and pressurized gas is pumped from a gage house to an opening in the tube. The hydrostatic pressure of the water determines the pressure reading inside the tube, so an increase in the water level translates to an increase in the hydrostatic pressure of the water and tube. The tube pressure is then converted to a stage value [11]. The bubble gage requires equipment housing, most often a look-in type of shelter that includes the pressure sensing equipment, a battery, an antenna for data transmission, and solar panels for battery charging. Both stilling wells and bubble gages are connected to gage houses that are equipped to collect stage data along with other parameters such as temperature, pH, and dissolved chemicals [11]. The downside of these stations is that they are large and permanent, prohibiting the transplant of a station to a more at-risk area. USGS stations are also expensive, limiting the number of gages established in a watershed and constraining the sampling density.

**Fig. 1:**
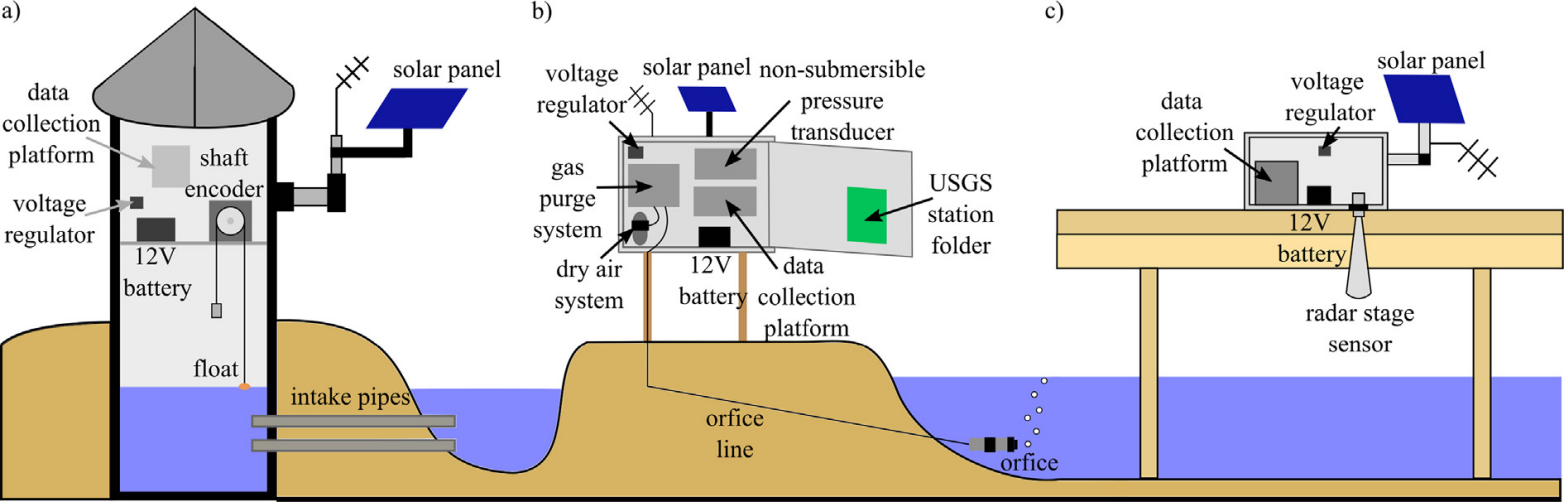
Types of USGS stage gages: a) stilling well, b) bubble gage with look-in housing, c) rapid-deployment gage.

Rapidly-deployable sensors, such as the USGS Rapid-Deployment Gages (RDGs), are deployed temporarily to measure hydraulic parameters in emergency situations. The USGS RDGs contain a radar-based stage sensor, radio transmission, and solar panels, and are manually installed by bolting brackets onto a bridge. These are considerably smaller and more portable than the permanent gages, but they are still large and require experts to install [12]. Installing one of these RDGs in an emergency situation poses safety issues for the person installing the station as they are heavy, somewhat difficult to install, and would have to be deployed manually in inclement weather. Diagrams of each USGS gage type can be seen in Fig. 1.

**Fig. 2:**
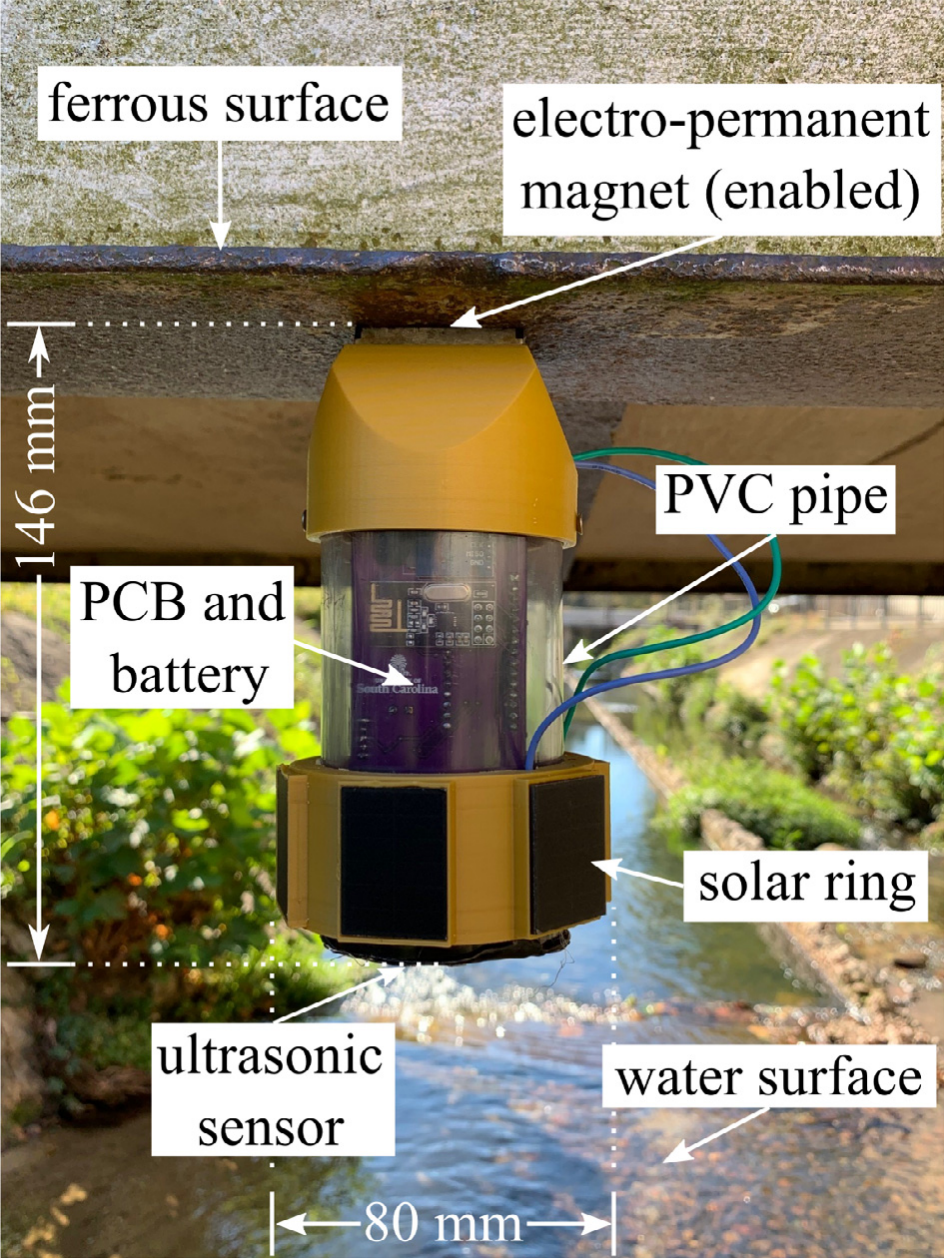
Stage sensor package deployed under a ferrous structure with solar add-on module..

The development of a Unmanned Aerial Vehicle (UAV)-deployable stage sensor package is reported in this work. As presented in Fig. 2, the UAV-deployable stage sensor package is designed to be mounted to the underside of ferrous structures using an electro-permanent magnet (EPM). The magnetic field of EPMs can be toggled on and off using electrical pulses [13], [14]. EPMs do not require constant power to remain enabled like an electromagnet, and are therefore ideal for long-term deployments. The stage sensor can be configured with modular add-on packages consisting of a solar module and a wireless control module for the remote activation/deactivation of the EPM.

The base package is the simplest package meant for manual deployments lasting no longer than 6 days. The solar add-on module is suitable for longer deployments, as the use of solar load sharing prolongs the package’s lifespan depending on the amount of available solar radiation. The wireless control add-on module is most ideal for UAV applications as it allows UAV deployment using wireless control of the EPM. The EPM can be enabled and disabled from up to 10 meters away from the user, which is optimal for structures out of reach for manual deployment. A drawback of the magnet module is that the radio frequency (RF) protocol consumes over twice the power of the base package, resulting in shorter deployments. It is important to note that the solar and wireless control add-on modules are built on top of the same base package hardware and can be combined in one form factor to extend the life of the package. Each package is capable of measuring stage, humidity, temperature, and ambient atmospheric pressure.

This paper begins with an overview of the stage sensor package’s hardware and software, including the design characteristics, sensing technology, and data collection protocols. A list of design files and materials is presented, along with build and operation instructions. The paper concludes with tests validating sensor accuracy, deployment longevity, and a field study. The base package is explored in depth throughout this work and is used in all validation tests. The solar and wireless control add-on modules are specifically mentioned in their individual sections.

## 2 Hardware Description

### 2.1 Overview

The stage sensor package was developed with the following design goals:

1. Cost under 300 USD per unit
2. Lightweight design suitable for UAV deployment
3. Multi-day deployments
4. Wide range of operating conditions regarding temperature, humidity, *etc*
5. Wireless control for EPM during UAV deployment
6. Produce comparable results to the USGS bubble gages
7. Battery power monitoring system

**Fig. 3:**
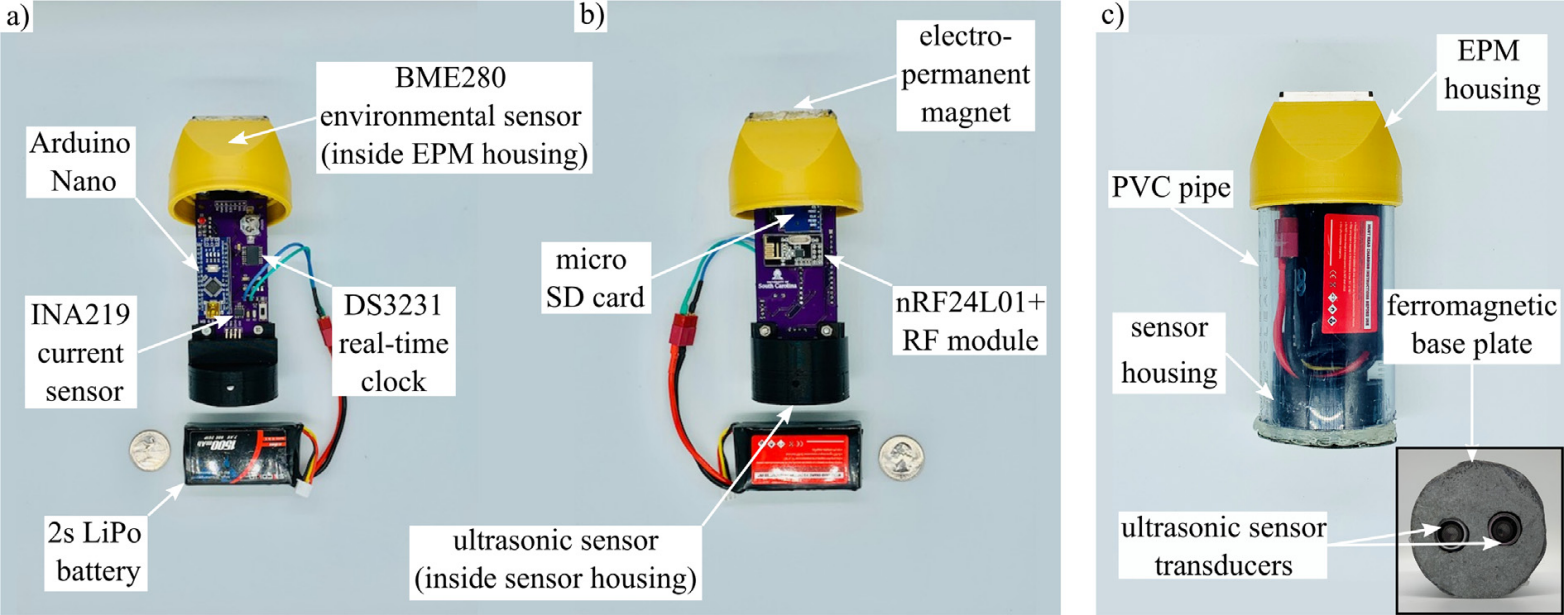
Internal components of sensor package a) front and b) back. The internal hardware is housed in the c) external assembly during deployment.

Fig. 3 shows the internal components of the sensor package. The most basic datalogger contains a microprocessor, time keeping device, and data storage device. The stage sensor package utilizes the Arduino Nano development board with an ATmega328P microcontroller. This particular microcontroller was chosen for its low power capabilities, many digital I/O pins, and SPI, I^2^C, and UART communication protocols. The 14 I/O pins allow for a large number of sensors to be included in the package, while the communication protocols facilitate fast, accurate sensor readings. A micro SD card module is featured to allow onboard data storage via SPI communication. The package employs the DS3231 real-time clock for time keeping, which uses I^2^C protocol to provide time stamps for sensor readings, allow consistent time intervals between collection cycles, and wake the microcontroller from its low power sleep mode. The DS3231 requires an external 3 V coin cell to provide power to the clock during sleep mode when the package is not being powered by the 7.4 V, 1500 mAh, lithium polymer battery.

To measure ambient environmental conditions, the BME280 environmental sensor was added. This sensor communicates temperature, humidity, and pressure readings via I^2^C. As the package is deployed outdoors, this sensor is useful for detecting conditions that may be associated with an incoming severe weather event. To avoid an overdischarged battery while the package is deployed, the INA219 current sensor was added to monitor the battery voltage, current, and power consumption. Table 1 gives an overview of the specifics of the sensor package.

**Table 1:** Design characteristics of stage sensor package.

Package type	Parameter	
Base package	Weight	0.38 kg
	Battery life	6.4 days
	Active current draw	18 mA
	Standby current draw	9 mA
	Stage range	0.02–3.5 m
Base package with solar add-on module	Weight	0.43 kg
	Battery life (ideal conditions)	13 days
	Max load sharing current	159.2 mA
	Active current draw	18 mA
	Standby current draw	9 mA
	Stage range	0.02–3.5 m
Base package with wireless control add-on module	Weight	0.38 kg
	Battery life	2.8 days
	Peak current draw during toggle	384 mA
	Active current draw	31 mA
	Standby current draw	22 mA
	Stage range	0.02–3.5 m
	RF range	10 m
Base package with solar add-on module & wireless control add-on module	Weight	0.43 kg
	Battery life (ideal conditions)	5 days
	Max load sharing current	159.2 mA
	Peak current draw during toggle	384 mA
	Active current draw	31 mA
	Standby current draw	22 mA
	Stage range	0.02–3.5 m
	RF Range	10 m

### 2.2 Ultrasonic Sensing

**Fig. 4:**
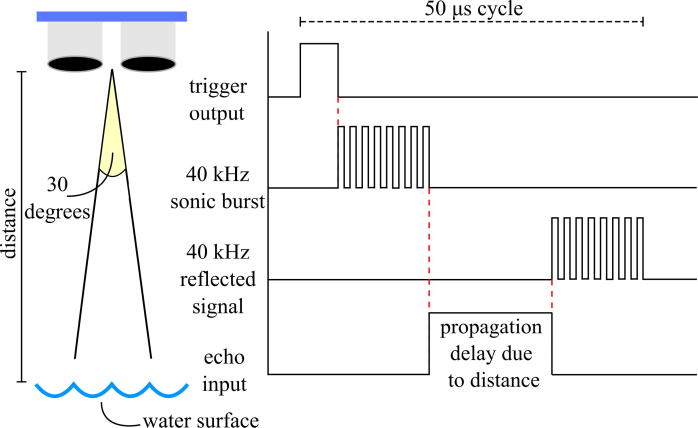
Timing diagram of HC-SR04 ultrasonic sensor.

The HC-SR04 ultrasonic sensor is used to measure the water level. The sensor uses a 40 kHz burst of ultrasound to measure the distance from the HC-SR04 at the base of the package to the water surface [15]. The stand-off measurement capability of the HC-SR04 made it an ideal choice for a UAV deployable package as it eliminates the need for submerged parts, which are required in the typical USGS bubble gages and stilling wells. Additionally, an ultrasonic sensor was chosen instead of a LiDAR sensor, another contactless method of measuring distance, due to LiDAR’s unsuitability for water applications [16]. The sensor has two transducers: the transmitter which converts the electrical signal from the microcontroller into an ultrasonic pulse, and the receiver that converts the reflected ultrasonic pulse back into an electrical signal to send to the microcontroller [17]. These two transducers are enclosed in metal cylinders on the sensor, which are exposed through two holes at the base of the stage sensor package. Fig. 4 outlines the timing diagram and working principle of the HC-SR04.

**Fig. 5:**
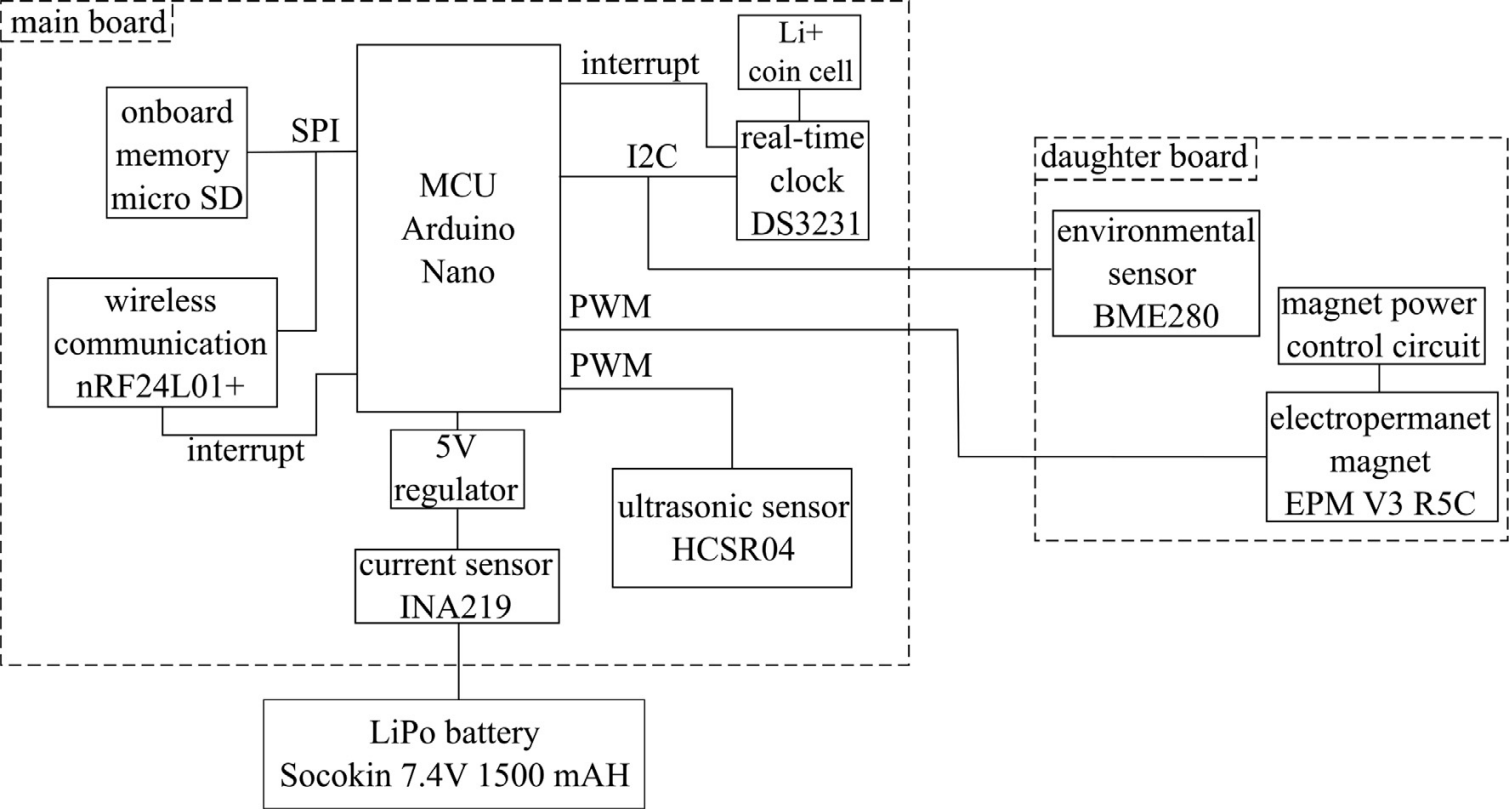
Block diagram of the stage sensor package.

The transducers in the HC-SR04 ultrasonic sensor have an effectual angle of 30 degrees [15], which is important as it constrains the distance a sensor package can be placed from any lateral obstructions, such as a bridge girder. For example, if the sensor package is placed at its maximum height of 4 m from the water surface, there may be no obstructions from the surface within a 1.07 m radius directly below the transducers of the HC-SR04. The transducers should be positioned parallel to the water’s surface to ensure the ultrasound reflects directly back toward the sensor. Once the microcontroller signals the HC-SR04 to take a distance reading, a 10 μs trigger output causes the transmitter to emit a 8 cycle 40 kHz ultrasonic burst. The receiver waits for the reflected 8 cycle 40 kHz signal, and when it picks up the same frequency, the echo pin of the sensor is driven high for the corresponding length of the delay between transmitter output and receiver input [17]. This signal is sent to the microcontroller and converted into a distance reading. It is important that each data collection cycle must be at least 50 μs long to avoid overlapping pulses. Fig. 5.

### 2.3 Power

Longevity is an important feature for the stage sensor package. The package has been optimized for maximum battery life, as it is uncertain how long a package should be deployed during a severe weather event. The ATmega328P has a sleep mode feature which allows for the power consumption to be reduced by cutting off all peripheral pins, including communication protocols, except for the interrupt pins. These interrupt pins will disable sleep mode when triggered, waking the microcontroller up for 6 s to record and save data, then allow the Arduino Nano to immediately resume sleep mode. One interrupt pin is attached to the real-time clock and is triggered every five minutes to run a data collection cycle. The second interrupt pin is wired to the nRF24L01 + to wake the microcontroller when a ping is sent from the RF controller to toggle the EPM in the wireless control module. It is important to note that although the ATmega328P is in sleep mode, there still exists steady state power consumption from the modules on the PCB.

### 2.4 Software

**Fig. 6:**
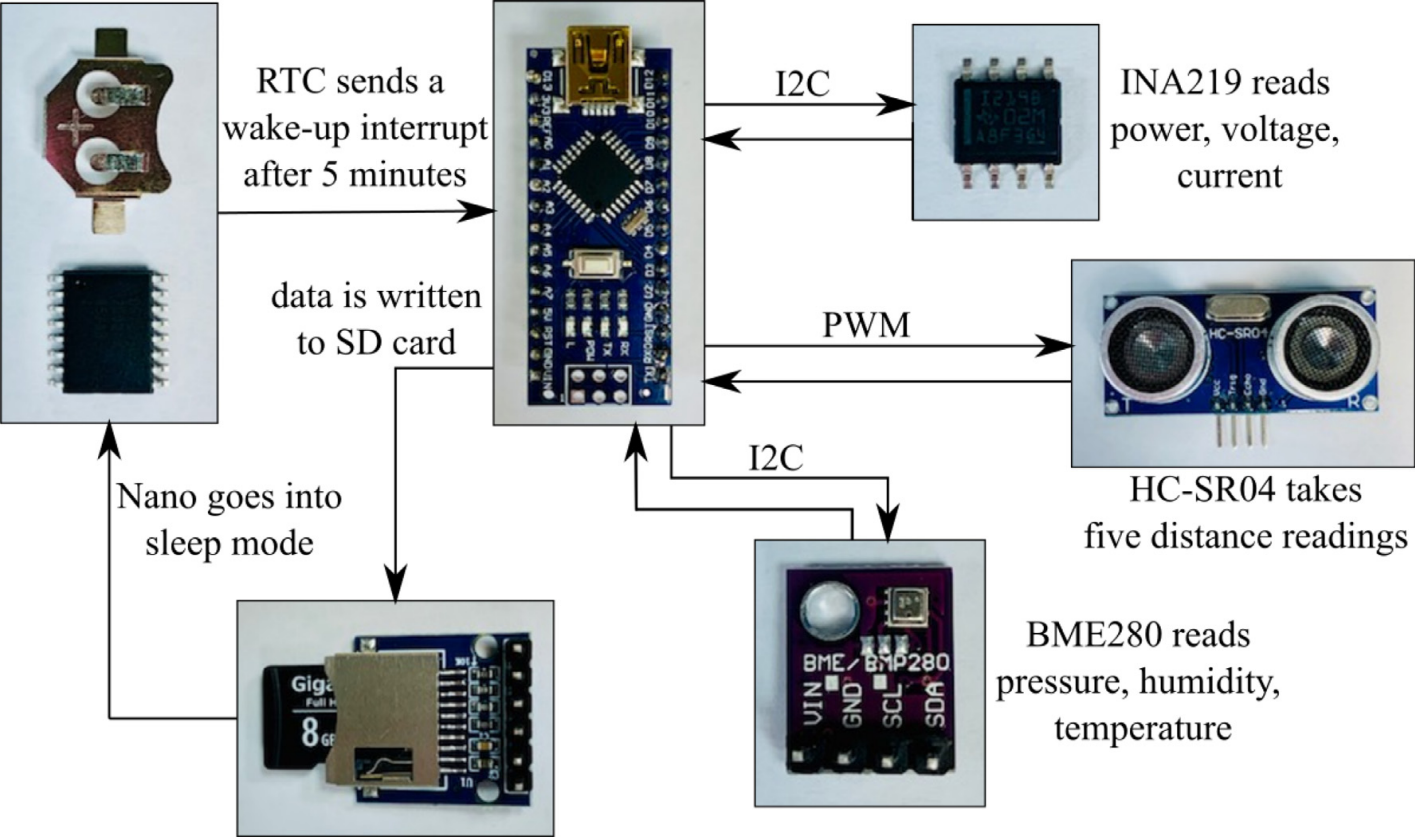
Algorithm for data collection and processing performed by the stage sensor package.

The basic data logging process of the stage sensor package is displayed in Fig. 6. The software of the stage sensor package is Arduino-language based. When first powered on, the ATmega328P configures all of the modules and then immediately sleeps for five minutes. The data collection process begins with the DS3231 sending an interrupt to wake up the microcontroller to execute the data collection and logging code. The HC-SR04 first takes five stage readings that are communicated to the microcontroller via PWM, and all non-zero distance measurements are averaged. The BME280 then measures temperature, pressure, and humidity and sends the readings via I^2^C to the ATmega328P. Finally, the INA219 reads the voltage, current, and power draw from the battery and also relays the values via I^2^C. Once all the sensor readings are taken, they are then written in a.csv file that is saved on the SD card using SPI, along with a time stamp of when the data was taken. After the data is written, another five-minute alarm is configured for the DS3231 before the ATmega328P is put back into sleep mode. When the alarm is triggered, the entire data collection process will repeat.

### 2.5 Solar Add-on Module

**Fig. 7:**
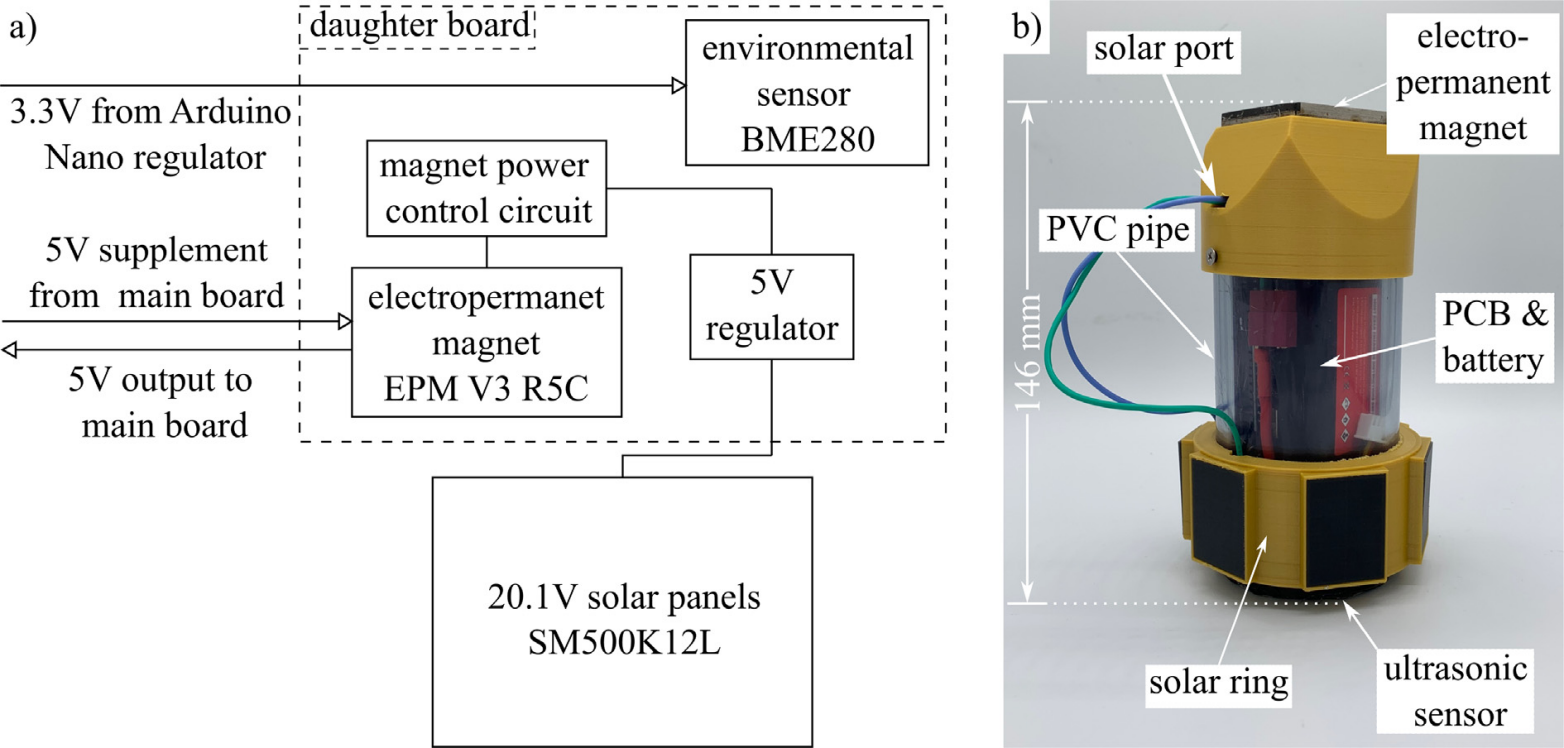
Solar module implemented on the base package. Solar load sharing is incorporated in the a) block diagram of the daughter board and b) external frame of solar package.

Load sharing with solar panels is an effective strategy to extend the deployment period of the package. The idea behind load sharing is that the package will draw power from the solar panels first, then supplement additional power needs with the 7.4 V LiPo battery. The solar cells are not recharging the battery, but rather acting as an alternative power source, meaning the 7.4 V LiPo battery should be recharged after deployment. Fig. 7 shows the block diagram of the solar add-on module and its outer frame. The 3D printed solar ring contains six chambers, each for one SM500K12L Anysolar 132.3 mW solar panel. The panels are configured so that three panels are wired in series, then two sets of three are wired in parallel. This allows for a maximum voltage of 20.1 V and current draw of 39.6 mA. The solar ring has a connector that is inserted through the solar port on the EPM housing and plugs into the daughter board. The voltage from the solar panels is stepped down with a 5 V regulator on the daughter board, which in turn steps up the current draw to a maximum of 159.2 mA.

In the field, the packages will likely be deployed under structures that block sunlight, so the power generated may be much less than the maximum, which is why solar panels that provide much more power than necessary for the package were chosen. Additionally, solar panels are light and compact, making them suitable for the UAV deployable design goal as opposed to alternative power sources.

### 2.6 Wireless Control Add-on Module

The wireless control add-on module allows the EPM to be controlled remotely. Contrary to the base package, which treats the EPM as a permanent magnet and is manually deployed, the wireless control module allows the EPM to be toggled on and off with an RF controller. This is useful for UAV deployment, as the user can attach and detach the package from a structure out of reach for manual deployment.

The wireless control module requires an RF controller to toggle the EPM. The controller can be paired with up to three different packages, making it suitable for deploying multiple packages in a network. The wireless control module uses RF communication via the nRF24L01 + 2.4 GHz wireless module located on both the controller and the package itself. The nRF24L01 + on the controller sends a ping to the nRF24L01 + on the package, which then signals the ATmega328P to supply power to the EPM and send a PWM signal to the magnet that will either toggle it on or off, depending on the ping sent from the controller. As the EPM does not need power after it is enabled and attached to the structure, a magnet power control circuit on the daughter board consisting of a high-side switch disconnects the 5 V trace to the EPM to avoid steady state power consumption.

**Fig. 8:**
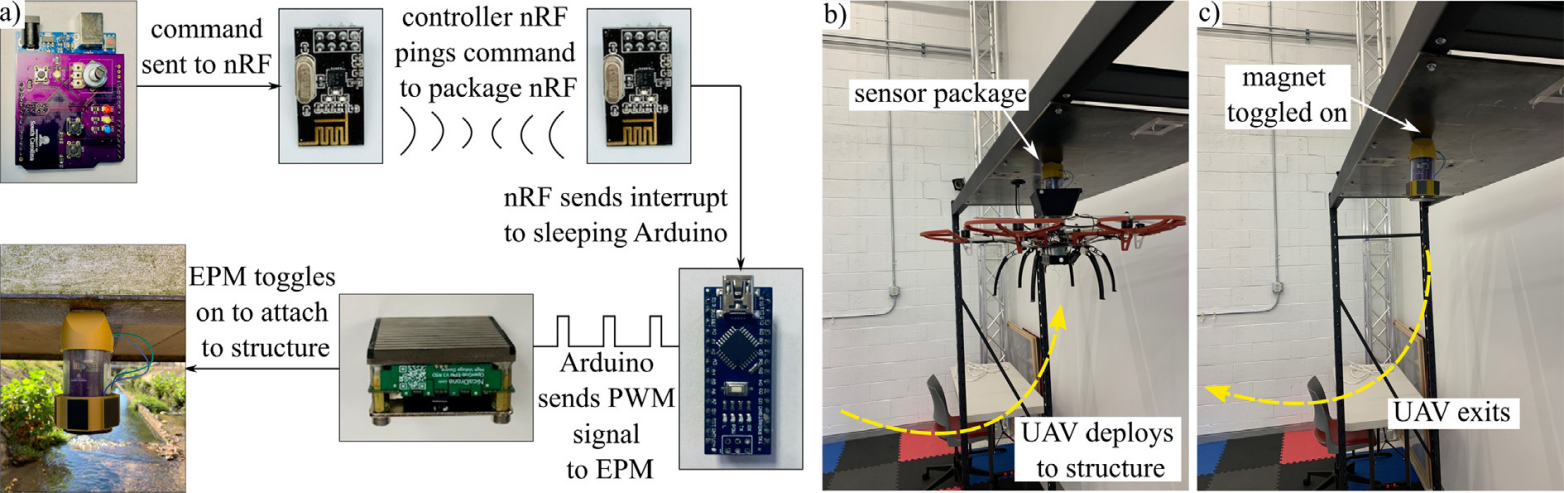
Wireless control module implemented on base package. RF communication enables the a) control sequence of wireless contorl module for use b) during UAV deployment and c) after fixation to structure.

Carrol et al. [14] explored a UAV deployment system with a vibration sensor package. This stage sensor package would be delivered in the same manner under bridges spanning bodies of water prone to flash flooding. The package will be flown out under a bridge with a ferrous surface that allows for magnetization, and the EPM on the package will be engaged to attach to the structure. Then, a second EPM on the UAV will disengage to release the package, leaving the package magnetized to the bridge to monitor stage. To retrieve the package, the UAV will fly up under the package and engage its EPM to the ferrous plate at the base of the package. The package EPM will then be disengaged from the bridge, allowing the UAV and package to fly away. This control sequence is outlined in Fig. 8.

## 3 Design Files Summary

Table 2 contains a list of all design files used in the base package, solar add-on module, and EPM add-on module. All files including STL files, PCB files, Arduino code, and video instructions can be located in the Open Science Framework repository at https://doi.org/10.17605/OSF.IO/A874U.

## 4 Bill of Materials Summary

A complete bill of materials is located in https://doi.org/10.17605/OSF.IO/A874U.

## 5 Build Instructions

This section outlines the build instructions for the base package, solar add-on module, and EPM add-on module.

### 5.1 Base package

The base sensor package is built in two stages. First, the PCB must be soldered with the proper electronic components. Second, the package housing consisting of the 3D printed parts, PVC pipe, and battery must be assembled. Tools used for the construction of the package include the following:

• Weller WES51 soldering iron
• MG Chemicals Sn60/Pb40 22 gauge solder
• MG Chemicals Sn63/Pb37 solder paste
• Phillips head screwdriver
• M2.5 Allen wrench
• Pliers
• Wire cutters/strippers
• Hot glue/epoxy

#### 5.1.1 Building the PCB

1. Begin by obtaining the main and daughter PCBs provided from the electronic design automation (EDA) files in Table 2.

**Table 2:** UAV-deployable stage sensor design files.

Design file name	File type	Open sourced license	Location of file
Main PCB	EDA file	CC-BY 4.0	https://doi.org/10.17605/OSF.IO/A874U
Daughter PCB	EDA file	CC-BY 4.0	https://doi.org/10.17605/OSF.IO/A874U
EPM housing	STL file	CC-BY 4.0	https://doi.org/10.17605/OSF.IO/A874U
Sensor housing	STL file	CC-BY 4.0	https://doi.org/10.17605/OSF.IO/A874U
Base sensor plate	STL file	CC-BY 4.0	https://doi.org/10.17605/OSF.IO/A874U
Data collection sketch	Arduino file	CC-BY 4.0	https://doi.org/10.17605/OSF.IO/A874U
Clock calibration sketch	Arduino file	CC-BY 4.0	https://doi.org/10.17605/OSF.IO/A874U
Magnet toggle sketch	Arduino file	CC-BY 4.0	https://doi.org/10.17605/OSF.IO/A874U
Solar ring	STL file	CC-BY 4.0	https://doi.org/10.17605/OSF.IO/A874U
RF controller PCB	EDA file	CC-BY 4.0	https://doi.org/10.17605/OSF.IO/A874U
EPM data collection sketch	Arduino file	CC-BY 4.0	https://doi.org/10.17605/OSF.IO/A874U
RF transmitter sketch	Arduino file	CC-BY 4.0	https://doi.org/10.17605/OSF.IO/A874U
Data headers	CSV file	CC-BY 4.0	https://doi.org/10.17605/OSF.IO/A874U
2. Beginning with the main board, solder the surface mount components at 400–425 °C using the solder paste. Make sure to note the resistor and capacitor values found in Table 3. A guide to the order and orientation of SMD components is given in Fig. 9.

**Table 3:** Selected SMD component values and PCB labels.

(a) Resistor values and labels.		
Label	Component	Board
R1	1 kΩ Resistor	Daughter PCB
R2	100 Ω Resistor	Daughter PCB
R3	100 Ω Resistor	Daughter PCB
R1	1 kΩ Resistor	Main PCB
R2	0.1 Ω Resistor	Main PCB
R3	4.7 kΩ Resistor	Main PCB
R4	4.7 kΩ Resistor	Main PCB
R5	4.7 kΩ Resistor	Main PCB
		
(b) Capacitor values and labels.		

Label	Component	Board

C1	0.1 μF Capacitor	Daughter PCB
C1	0.1 μF Capacitor	Main PCB
C2	0.1 μF Capacitor	Main PCB
C3	0.1 μF Capacitor	Main PCB
C4	0.1 μF Capacitor	Main PCB

**Fig. 9:**
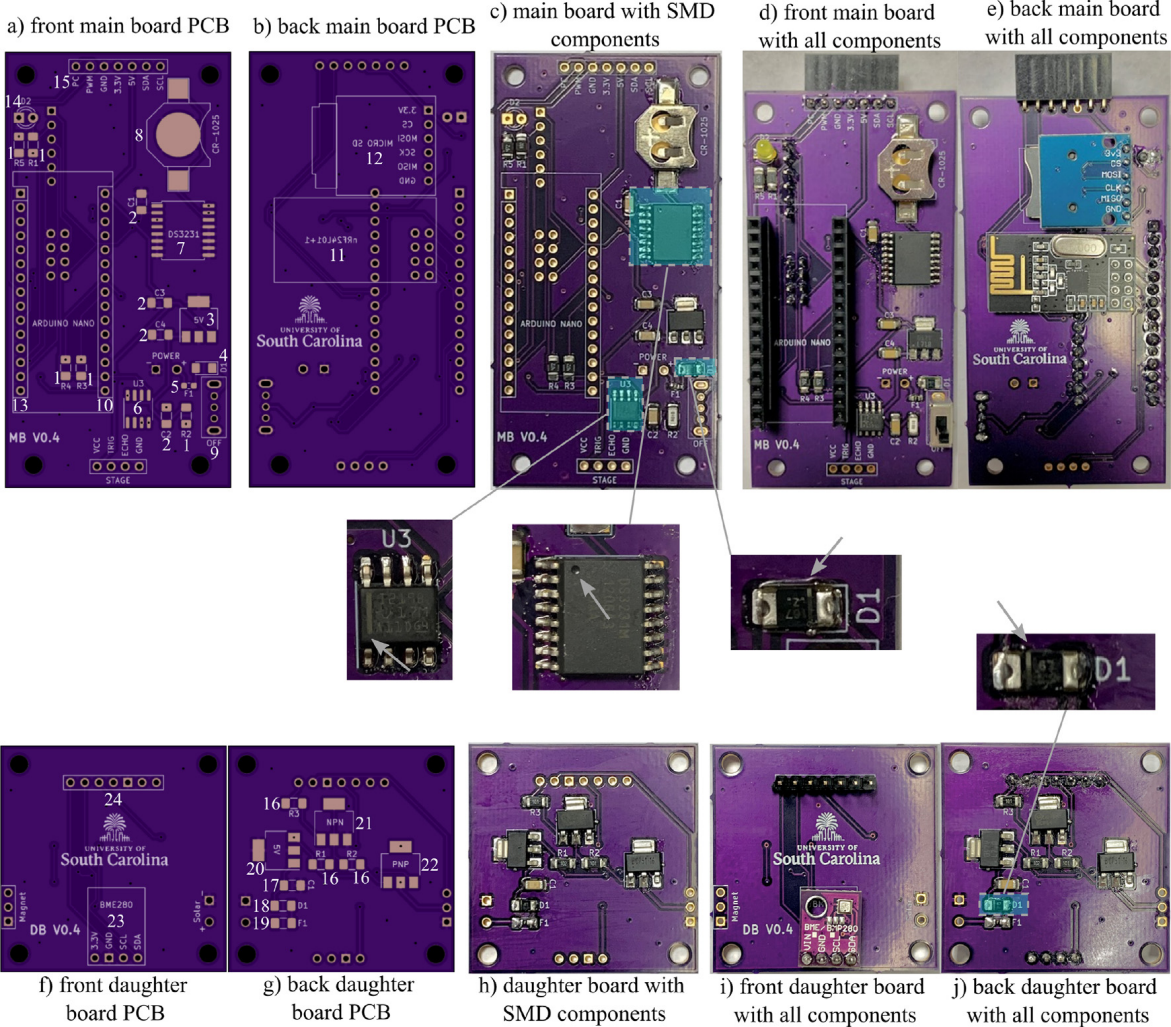
Soldered components on main and daughter PCBs. Numbers denote corresponding parts in the PCB # column in the bill of materials and suggested order of soldering.
3. Once the surface mount components have been soldered, begin soldering the through hole components with the 22 gauge solder.
4. As the PCB is double sided, in order to facilitate the through hole soldering, the following order of components is recommended: left Arduino rail, LED, nRF24L01+, micro SD card module, right Arduino rail, slide switch, female daughter board connector. Do not solder the female HC-SR04 connector or power port yet. The suggested order of soldering is presented in Fig. 9.
5. During the soldering process, use the wire cutters to cut any through hole leads that extend far out from the PCB to avoid creating unwanted contacts.
6. Solder one end of a 130 mm black wire to the negative contact of the Dean’s connector, and solder one end of a 130 mm red wire to the positive contact of the Dean’s connector.
7. Cut a 20 mm piece of black heat shrink tubing and a 20 mm piece of red heat shrink tubing and slide each piece over the corresponding wire color.
8. Heat up the tubing so that it molds around the junction between the wire and Dean’s connector.
9. Solder the other end of each wire into the corresponding power ports.
10. Moving on to the daughter board, solder the surface mount components using the solder paste. Make sure to note the resistor and capacitor values found in Table 3.
11. Once the surface mount components have been soldered, begin soldering the through hole components with the 22 gauge solder. DO NOT solder the female magnet connector or the solar power port yet.

#### 5.1.2 Building the outer frame

**Fig. 10:**
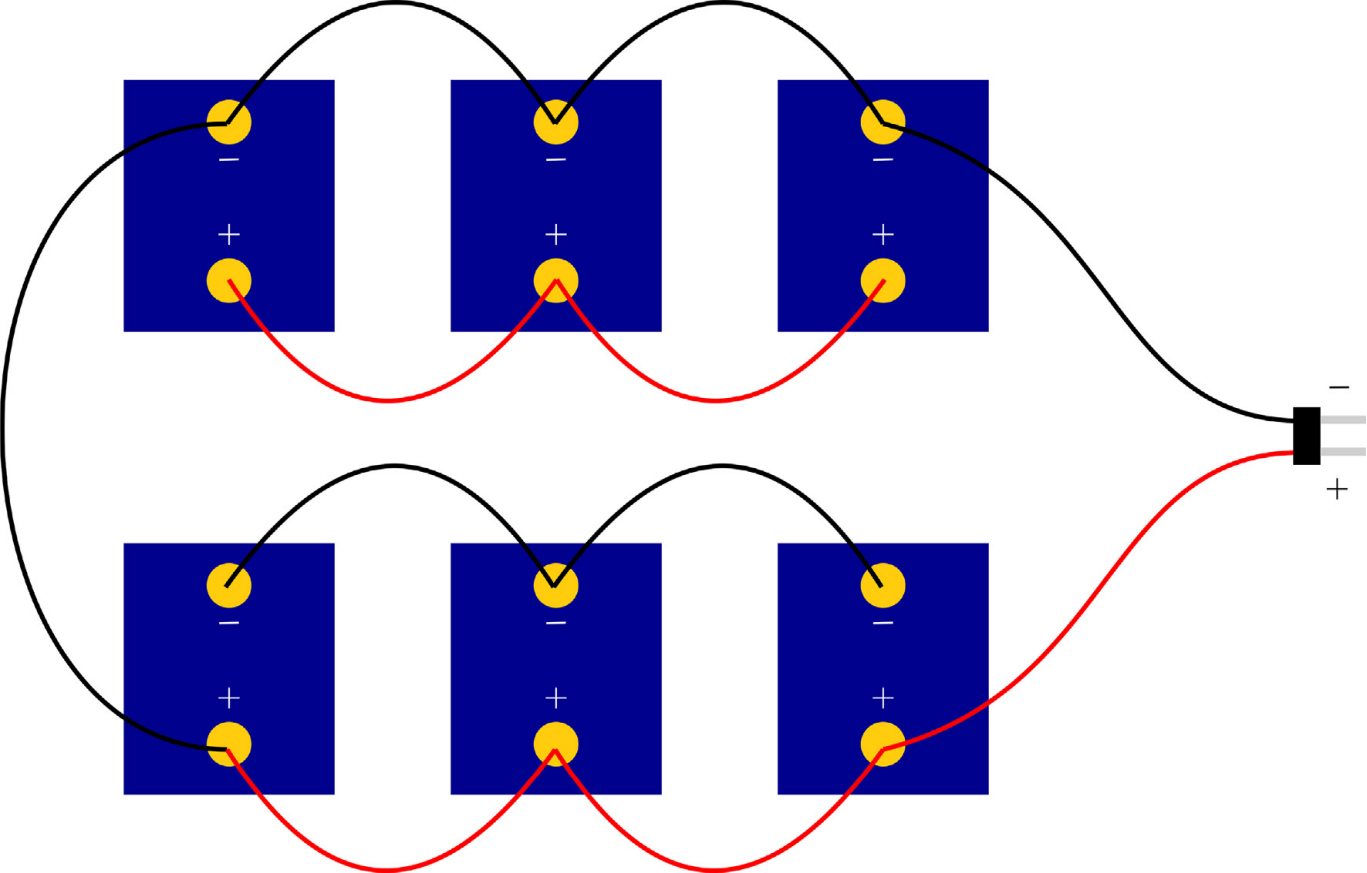
Wiring orientation for solar panels in solar ring. Panels are soldered with two series groups of three panels in parallel.

A video tutorial for building the base package can be found at https://doi.org/10.17605/OSF.IO/A874U. Fig. 10.

1. 3D print the sensor housing, EPM housing, and base plate Fusion 360 files listed in Table 2.
2. Spray all printed parts with workable fixatif to make them water resistant. For more waterproofing, the exterior of parts can be coated with marine epoxy.
3. Using pliers, bend the HC-SR04 contacts 90 degrees away from the transducer side and place the ultrasonic sensor inside the 3D printed sensor housing with the contacts facing upwards toward the slit in the top.
4. Place the main PCB in the top slot on the sensor housing, lining up the holes in the slot with the PCB. Screw the main PCB to the sensor housing using M3 screws.
5. Use the pliers to bend the female HC-SR04 connector 90 degrees so that the leads fit into the four through holes at the base of the PCB labeled STAGE. Make sure the connector reaches the ultrasonic sensor in the housing. Cut off any excess using the wire cutters.
6. Remove the female connector socket from the ultrasonic sensor and solder to the main PCB.
7. Place the EPM over the daughter PCB, lining up the screw holes. Also place the three pin female magnet connector socket over the three male pins on the EPM.
8. Using the pliers, bend the connector sockets towards the port labeled MAGNET on the daughter PCB so that the socket leads reach down through the holes. It is important that the connector stays as tightly within the boundary of the EPM-PCB complex, so that it will fit properly within the EPM housing.
9. Once the connector is bent, solder the connector into the MAGNET port on the PCB, cutting off any excess with the wire cutters.
10. Connect the EPM at the socket and place the EPM-PCB complex flat in the EPM housing. Secure the complex with 4 M3 screws to the EPM housing.
11. Connect the top female sockets of the main PCB to the male pins on the daughter PCB, sliding the PCB through the guide slots on the magnet module.
12. Connect the ultrasonic sensor in its housing to the STAGE port on the bottom of the main PCB. Screw the main PCB to the sensor housing using M3 screws.
13. Cut a 105 mm piece of 2 in PVC pipe and drill two 3.2 mm holes at the top to align with the EPM housing.
14. Slide the entire assembly consisting of the sensor housing, PCB, and EPM housing into the PVC pipe and screw the EPM housing into the PVC.
15. Adhere the base plate to the bottom of the PVC pipe so that the holes align with the ultrasonic sensor transducers. NOTE: When using the EPM module for UAV deployment, replace the 3D printed base plate with a ferrous base plate of the same dimensions.

### 5.2 Solar Add-on Module

**Fig. 11:**
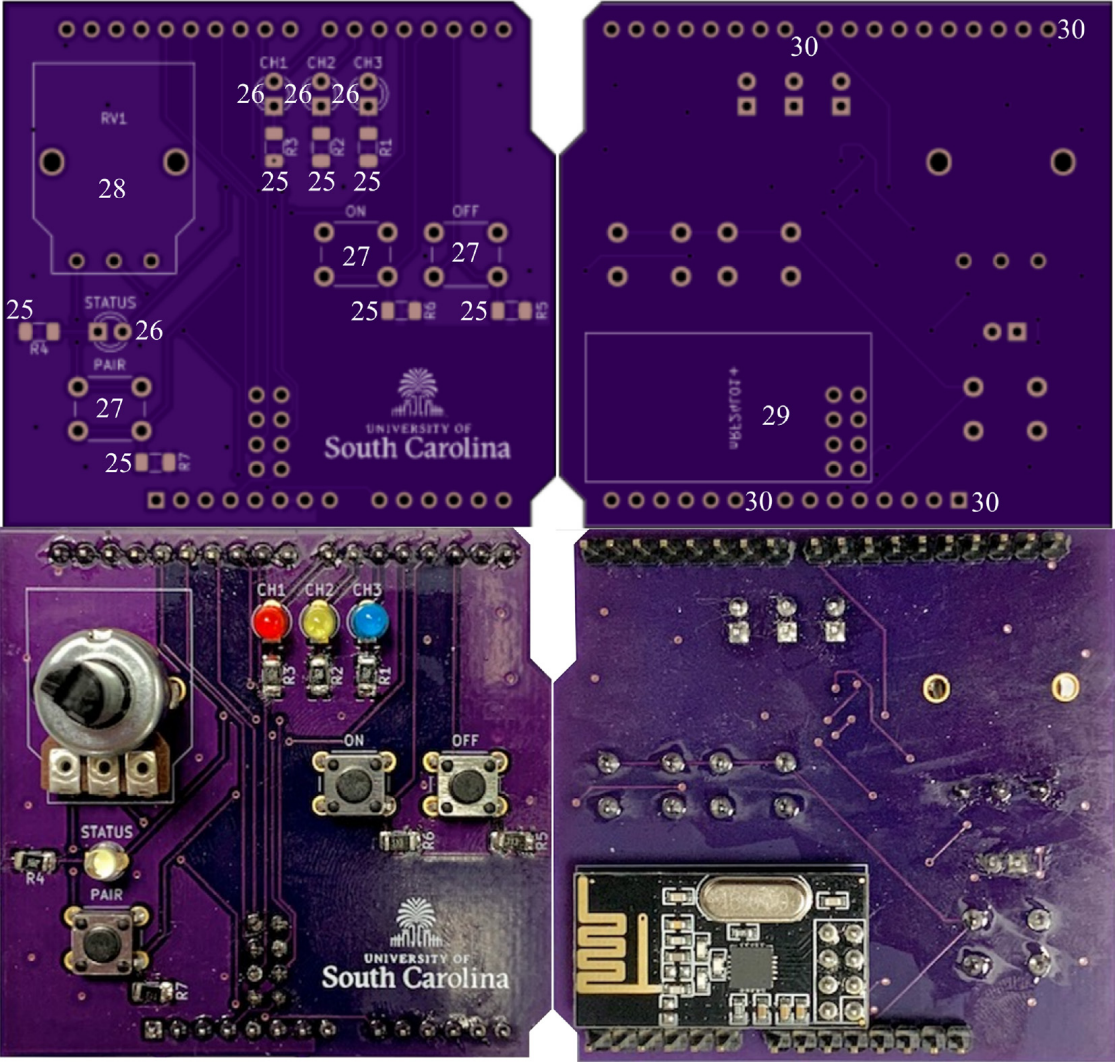
Soldered components on RF controller PCB. Numbers denote corresponding parts in the PCB # column in the bill of materials and suggested order of soldering.

The solar module allows load sharing with solar panels. This extends the life of the package depending on the environmental conditions by allowing the electronics to draw power from solar panels instead of the LiPo battery. Fig. 11.

1. Build the base package according to Section 5.1.
2. 3D print the solar ring Fusion 360 file from Table 2.
3. The six solar cells will be wired in series and parallel to maximize voltage and current draw. Three panels will be wired in parallel, then two sets of three will be wired in series.
4. Begin by soldering one 20 mm wire to the  + terminal of one solar cell and one 50 mm wire to the - terminal.
5. Thread the wires through the holes in the panel spot with the small divot at the top of the ring. Orient the panel so that the  + terminal is closest to the divot.
6. Use 50 mm wire to connect the next two solar cells in parallel with the first one, moving onto the adjacent spot without a divot at the top. The end result should be three solar panels wired in parallel.
7. Repeat the process beginning at the second divot, but instead wire the 200 mm wire to the - terminal of the solar panel and the 50 mm wire to the  + terminal.
8. Orient the - terminal of the solar panel closest to the divot, then wire two more solar panels in parallel in the remaining two slots as previously described.
9. The two sets of parallel solar panels should meet directly across from the divots. Here, wire the  + terminal of one of the unwired solar cells to the - terminal of the other. Then wire the - terminal to the  + terminal.
10. The result should be two sets of three parallel solar panels wired in series.
11. Secure the wires inside the ring to prevent damage.
12. Slide the solar ring over the top of the PVC pipe, allowing the two 200 mm wires to hang out of the divots.
13. Next, solder a two prong male header into the bottom of the solar port on the daughter board, so that the leads are sticking out on the same side as the BME280.
14. Solder the female solar connector onto the 200 mm wires of the solar ring.
15. Thread the connector through the solar port and connect the solar ring to the daughter board to allow for load sharing. NOTE: In order for load sharing to work, the solar panels must be connected before the battery. This way, they are the primary power source and the battery is supplementary.
16. Hot glue the connector into the solar port to prevent water leakage through the hole.

### 5.3 Wireless Control Add-on Module

The EPM module follows the exact same build instructions as the base package, with the addition of building a RF controller for toggling the EPM.

1. Build the base package according to Section 5.1.
2. Obtain the RF controller PCB provided from the KiCad files in Table 2.
3. Solder all surface mount components using the solder paste. The resistor values are as follows:
  • R1, R2, R3, R4: 1 kΩ
  • R5, R6, R7: 10 kΩ
4. Solder the 10 kΩ rotary potentiometer into the RV1 footprint. This will act as the channel switch.
5. Solder three LEDs into each of the channel footprints, and one LED into the STATUS footprint. The channel LEDs will indicate which sensor package the controller is communicating with, and the status LED will indicate whether or not there is a successful pairing.
6. Solder three button switches into the ON, OFF, and PAIR footprints. These will be the control buttons that determine which ping to send to the sensor package.
7. Solder the nRF24L01 + into its footprint. This is required for RF communication with sensor packages.
8. Solder male pin headers into the through holes lining the sides of the PCB. The pins allow the controller to act as a shield and connect on top of an Arduino Uno.
9. Attach the controller PCB as a shield on top of an Arduino UNO.
10. Connect a 9 V battery to the barrel jack of the Arduino UNO.

## 6 Operation Instructions

This section outlines the operation instructions for the base package, solar add-on module, and EPM add-on module. Video tutorials of all packages are available at https://doi.org/10.17605/OSF.IO/A874U.

### 6.1 Base Package

The base package is configured in two phases, beginning with the programming of the Arduino’s real-time clock, EPM, and data collection sequence. After the package software is set up, the outer frame is assembled and deployed under a ferrous structure.

#### 6.1.1 Programming the Arduino

1. Begin by removing the sensor housing from the PCB in order to access the USB port of the Arduino Nano (for reference on the correction orientation of the Nano, see Fig. 3).
2. Insert the Gigastone 8 GB micro SD card into the SD card module.
3. Insert the 3 V coin cell into the real-time clock battery holder to supply external power for the integrated circuit.
4. To set the real-time clock, upload the clock calibration sketch from Table 2. This will set the DS3231’s time to your computer’s time.
5. To toggle the magnet, upload the magnet toggle sketch from Table 2 to the Arduino Nano.
6. Place a ferrous object (such as sheet metal) over the EPM surface and use the serial monitor to send the appropriate command to toggle the magnet on.
7. Finally, upload the data collection sketch to the Nano.

#### 6.1.2 Deploying the package

1. Ensure the package is built according to the outer frame build instructions.
2. Ensure the power switch is in the OFF position before plugging in the 2s LiPo battery.
3. Once the battery is in place, flip the switch to the ON position. Note that the package will sleep for 5 min before taking the first sensor measurement.
4. Slide the PCB assembly and battery into the PVC pipe with the sensor housing first. Take care that all wires are inside the pipe and no wires are in danger of causing a short circuit.
5. Screw in the sides of the magnet module into the outer pipe using M3 screws.
6. A ferrous surface is required for the EPM to attach. At the desired gaging location, attach the engaged EPM face against a ferrous surface with an area larger than the EPM on the structure.
7. After sufficient data is collected, remove the package from the structure and disassemble.
8. Flip the power switch to the OFF position and unplug the LiPo
9. Remove the SD card and read the data using the computer. NOTE: the data will show up as raw values, match each value to its corresponding header using the data headers CSV file.

### 6.2 Solar Add-on Module

1. To enable solar load sharing, first complete Section 6.1.1 to program the Arduino.
2. Slip the solar ring around the top of the PVC pipe, with the connector facing upwards.
3. If not previously completed, thread the connector of the solar ring through the solar port on the EPM housing and plug into the port on the daughter board.
4. After the solar load sharing is plugged in, follow the steps in Section 6.1.2.
5. After unplugging the LiPo, unplug the solar ring.
6. Remove the SD card and read the data using the computer. NOTE: the data will show up as raw values, match each value to its corresponding header using the data headers CSV file

### 6.3 Wireless Control Add-on Module

1. Begin by removing the sensor housing from the PCB in order to access the USB port of the Arduino Nano (for reference on the correction orientation of the Nano, see Fig. 3).
2. Insert the Gigastone 8 GB micro SD card into the SD card module.
3. Insert the 3 V coin cell into the real-time clock battery holder to supply external power for the integrated circuit.
4. To set the real-time clock, upload the clock calibration sketch from Table 2. This will set the DS3231’s time to your computer’s time.
5. Upload the EPM data collection sketch to the package.
6. Upload the RF transmitter sketch to the assembled RF controller.
7. Ensure the package is built according to the outer frame build instructions (Reference Fig. 3).
8. Ensure the power switch is in the OFF position before plugging in the 2s LiPo battery.
9. Once the battery plugged in, flip the switch to the ON position. Note that the package will sleep for 5 min before taking the first sensor measurement.
10. Slide the PCB assembly and battery into the PVC pipe with the sensor housing first. Take care that all wires are inside the pipe and no wires are in danger of causing a short circuit.
11. Screw in the sides of the EPM housing into the outer pipe using M3 screws.
12. Use the potentiometer on the RF controller to choose the channel that corresponds to the desired package.
13. Press the pair button to ensure communication between the package and controller. If communication is successful, the status LED will turn on.
14. To deselect a channel and pair with a different channel, press the pair button again to turn off the status led and allow a new channel to be selected. Pair to the new channel.
15. A ferrous surface is required to attach the EPM. Once the correct package is paired, place the magnet module against the point of deployment on the structure surface.
16. Press the ON button on the RF controller to enable the EPM. The package should now adhere to the structure.
17. After sufficient data is collected, remove the package by pressing the OFF button on the RF controller. Note that the magnet will immediately turn off, so ensure the package is properly handled and will not fall off the structure into the water body below.
18. Remove the screws and lift the PCB assembly and battery from the tube.
19. Flip the power switch to the OFF position and unplug the LiPo
20. Remove the SD card and read the data using the computer. NOTE: the data will show up as raw values, match each value to its corresponding header using the data headers CSV file

## 7 Validation and Characteristics

### 7.1 Distance Validation

**Fig. 12:**
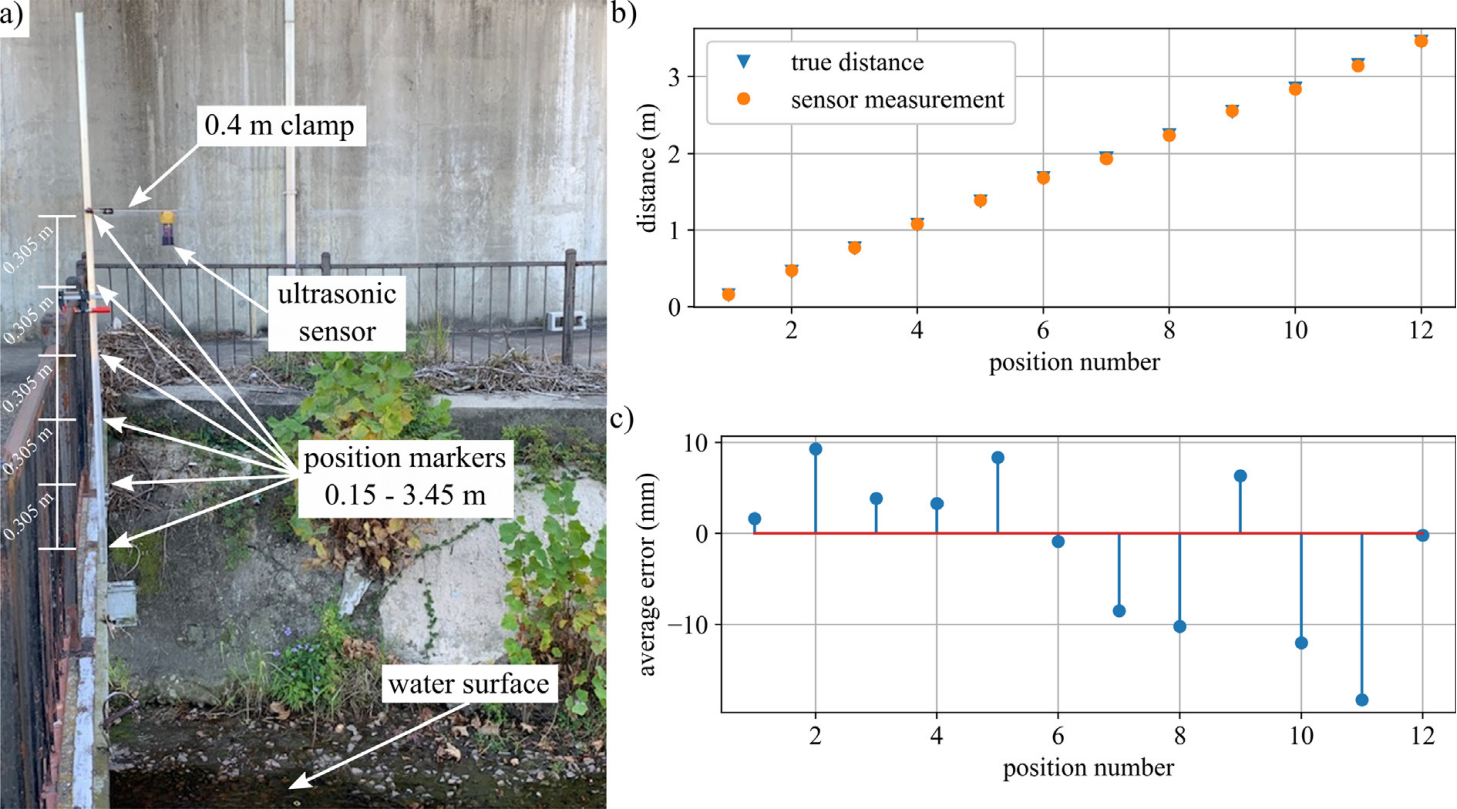
Distance validation test for HC-SR04 in base package. Shown is the a) experimental setup, b) sensor versus true distance comparison at each position, and c) average error at each position.

The accuracy of the ultrasonic sensor was tested in the distance study discussed here. The package was attached to a pole with a 0.4 m C–clamp and situated directly over a creek, where it took recordings at 12 different known distances spaced 0.305 m apart ranging from 0.15 to 3.45 m. The package offset was accounted for and subtracted from each position’s distance to get the true distance of the ultrasonic sensor from the water surface. The offset was calculated to be approximately 0.19 m. Given the HC-SR04’s effectual angle of 30 degrees, a 0.4 m clamp allows the package to be placed up to 1.49 m above the bridge before the ultrasound reflects off the structure. Fig. 12 shows the experimental setup and results.

After accounting for the offset of the package, the true distance was plotted against the average sensor reading at each position. The average error of the readings is 6.9 mm with a standard deviation of 0.89, and error magnitude increases slightly with increased distance. The source of the error could be due to the neglected deflection of the cantilever beam. The HC-SR04 has a documented resolution of 3 mm [17], so the error could originate from the hardware itself.

### 7.2 Longevity validation

The deployment length of the stage sensor was tested in the longevity study. In this validation test, the base package was examined. Before beginning the study, the theoretical deployment length of a package was calculated. The package draws 18 mA when taking readings for approximately 6 s, then 9 mA during sleep mode for the remaining 294 s in a five minute cycle. The LiPo battery used to power the package is a 2 cell 7.4 V 1500 mAh battery. Using current draw and battery capacity, the theoretical longevity of the package can be calculated as 6.8 days:

(1) $$\mathrm{Active}:18\mathrm{mA}\times 6s\times \frac{1\mathrm{hr}}{3600s}=0.03\mathrm{mAh}$$

(2) $$\mathrm{Sleep}:9\mathrm{mA}\times 294s\times \frac{1\mathrm{hr}}{3600s}=0.735\mathrm{mAh}$$

(3) $$1500\mathrm{mAh}\times \frac{5\min}{0.735\mathrm{mAH}+0.03\mathrm{mAh}}\times \frac{1\mathrm{hr}}{60\min}=163.4\mathrm{hr}=6.8\mathrm{days}$$

The experimental longevity of the package was determined via a benchtop test in which the fully-charged LiPo was plugged into the package and allowed to take readings for fixed five minute intervals until the battery voltage dropped below 7.4 V, at which point a buzzer would go off indicating the battery has been drained. The package was in a controlled environment at room temperature for the entire duration of the test.

**Fig. 13:**
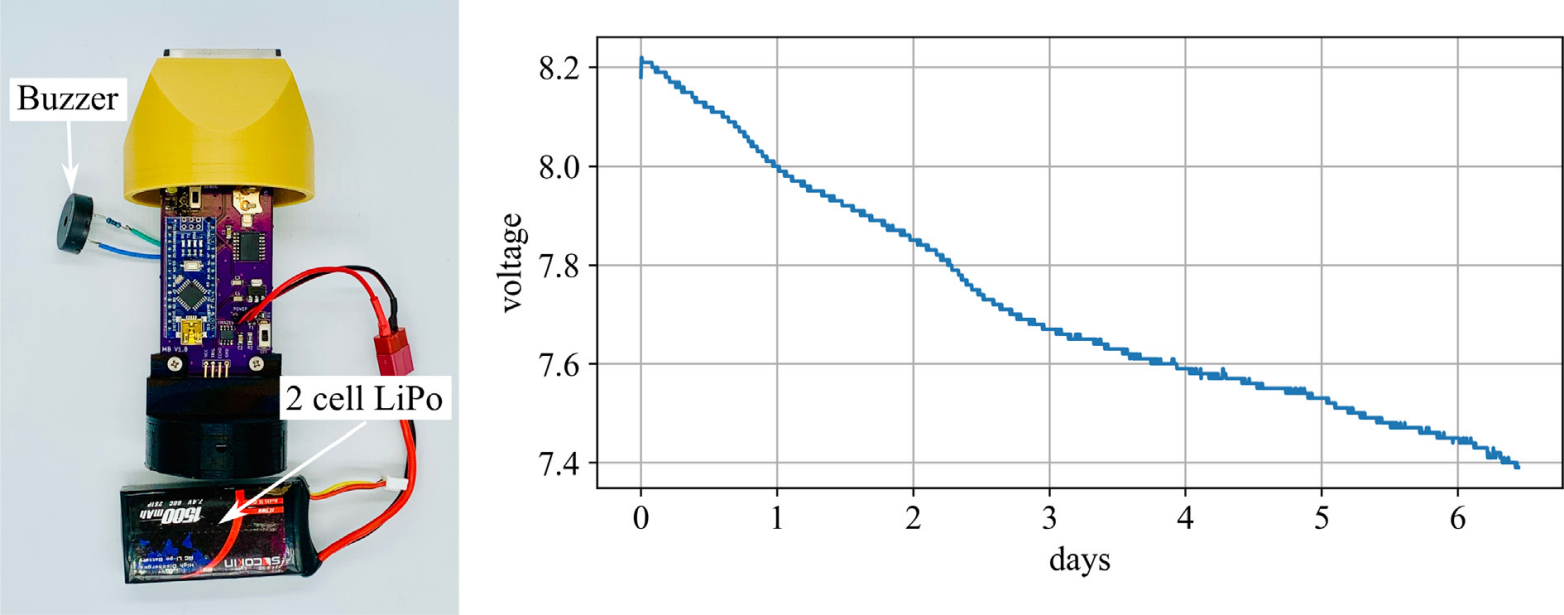
Experimental setup and voltage drop of fully-charged 2 cell LiPo over testing period.

Fig. 13 shows the voltage decay of the LiPo during the testing period. The battery was charged to 8.18 V and was drained to 7.39 V over a period that lasted 6.4 days. The experiment shows that the package is capable of lasting for 94.1% of the calculated 6.8 day lifespan, allowing for long lasting deployments in a typical application.

### 7.3 Field Study

The USGS stage gages are required to have an accuracy of either 3.05 mm or 0.2% of the stage being measured, depending on which value is greater [9]. As previously stated in Section 7.1, the stage sensor package has an average error of 6.9 mm, which is slightly greater than the smallest required tolerance of the USGS gages. Additionally, USGS gages measure stage at fixed intervals ranging from 5 to 15 min long. These instantaneous values are processed later, much like the post-processing nature of the UAV-deployable stage height sensor package. Considering the package collects data in a similar manner and resolution as the USGS gage, a field test was conducted to determine the viability of the package as a more rapidly-deployable stage sensor alternative to the USGS gages.

**Fig. 14:**
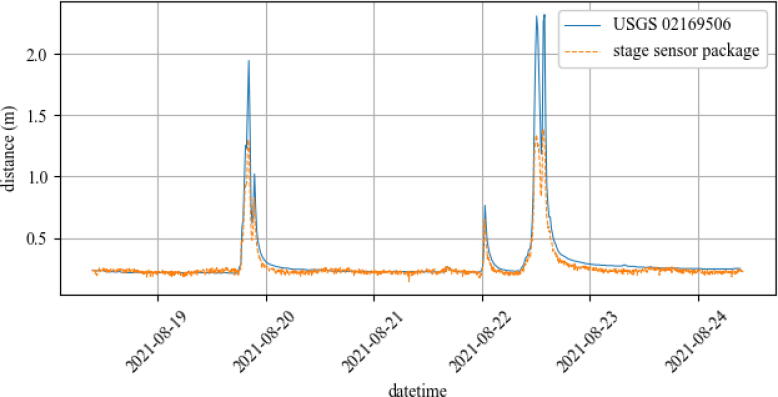
Field test data of UAV-deployable stage height sensor package compared to USGS Whaley St. Station data during subsequent rain events.

The sensor package was manually deployed on a ferrous bridge over the course of six days. The bridge is located on the Rocky Branch creek within 240 meters of the USGS 02169506 Rocky Branch at Whaley St. gaging station in Columbia, South Carolina. Differences in channel width and topography will affect the amplitude of the recorded stage, however a rainfall event should still produce the same number and frequency of peaks in stage for both the stage sensor package and the USGS gage. The results from the six day deployment are shown below in Fig. 14.

The stage sensor package measures distance from the HC-SR04 to the water surface. The distance between the HC-SR04 to the zero point defined by the USGS was measured to be 2.35 m. Stage readings must therefore be mirrored in order to measure vertical distance from the defined zero point to the water surface rather than from the HC-SR04 to the water surface. Fig. 14 shows the post-processed package stage readings measured from the fixed zero reference point.

The stage sensor was able to detect each peak that the USGS gage detected, as well as measure the water’s return to the same zero point between rainfalls. The amplitudes of each peak recorded by the stage sensor package are significantly lower, which is likely due to different channel profiles at the gaging locations. The channel was slightly wider at the deployed stage sensor than at the USGS gage, which resulted in a lower magnitude of peak stage during rain events. In this case, the package’s ability to measure each peak in stage is the best indicator that it is able to record rainfall event data to a similar competency as the USGS stage gage.

## 8 Conclusion

The stage sensor package described in this work will be a useful tool in analyzing flood trends and monitoring possible damage caused by past flood events. The low-cost nature of the package and easily available hardware allows for multiple packages to be constructed and deployed within a single watershed. Increased sampling density will provide a clearer record of flood frequency, magnitude, duration and volume within a region, allowing better conclusions to be drawn about how flooding in an area is changing. Auxiliary storage allows large amounts of data to be saved during each deployment, providing a more expansive record of past flood events, particularly nuisance flooding, that can be used to assess problem areas and take precautions. The contactless distance measurement method keeps each package compact and reduces the environmental requirements for deployment zones. The solar modification increases the package’s longevity and allows more data to be captured, and the EPM modification permits deployment in areas where manual deployment is not feasible.

Some major limitations of the design are longevity and data transmission. In order to increase the longevity of the package, a solar recharging circuit, as opposed to a solar load sharing circuit, should be implemented. Additionally, an adaptive sampling algorithm could reduce the frequency of data collection if there is no change in stage conditions, thus lowering power consumption. Concerning data transmission, the current design stores data with onboard memory and is suitable for recording past flood events, but if a rapid-response system that required real-time monitoring was needed, data transmission would need to be integrated.

**Human and animal rights**

No human or animal studies were conducted in this work.b

## CRediT authorship contribution statement

**Corinne Smith:** Investigation, Software, Methodology, Validation, Visualization, Writing - original draft. **Joud Satme:** Investigation, Methodology, Validation. **Jacob Martin:** Investigation, Validation. **Austin R.J. Downey:** Conceptualization, Methodology, Funding acquisition, Project administration, Writing - review & editing. **Nikolaos Vitzilaios:** Resources, Writing - review & editing. **Jasim Imran:** Supervision, Funding acquisition, Writing - review & editing.

## Declaration of Competing Interest

The authors declare that they have no known competing financial interests or personal relationships that could have appeared to influence the work reported in this paper.
